# Chlorogenic Acid Inhibits the Replication and Viability of Enterovirus 71 *In Vitro*

**DOI:** 10.1371/journal.pone.0076007

**Published:** 2013-09-30

**Authors:** Xiang Li, Yuanyuan Liu, Xueling Hou, Hongjun Peng, Li Zhang, Qingbo Jiang, Mei Shi, Yun Ji, Yuyue Wang, Weifeng Shi

**Affiliations:** 1 Department of Clinical Laboratory, The Third Affiliated Hospital of Suzhou University, Changzhou, Jiangsu, China; 2 Department of Endocrinology, The the Huai-an First Affliated Hospital of Nanjing Medical University, Huaian, Jiangsu, China; St. Jude Children's Research Hospital, United States of America

## Abstract

Enterovirus 71 (EV71) is an etiology for a number of diseases in humans. Traditional Chinese herbs have been reported to be effective for treating EV71 infection. However, there is no report about the antiviral effects of CHA against EV71. In this study, plaque reduction assay demonstrated that the inhibitory concentration 50% (IC50) of CHA on EV71 replication is 6.3 µg/ml. When both CHA (20 µg/ml) and EV71 were added, or added post-infection at different time points, CHA was able to effectively inhibit EV71 replication between 0 and 10 h. In addition, CHA inhibited EV71 2A transcription and translation in EV71-infected RD cells, but did not affect VP1, 3C, and 3D expression. Furthermore, CHA inhibited secretions of IL-6, TNF-α, IFN-γ and MCP-1 in EV71-infected RD cells. Altogether, these results revealed that CHA may have antiviral properties for treating EV71 infection.

## Introduction

Enteroviruses belong to the family Picornaviridae, which has >70 serotypes including poliovirus (types 1, 2 and 3), coxsackievirus A (A1-22 and A24), coxsackievirus B (B1–6), echovirus (1–21 and 24–33) and enterovirus (68–71, 73–78 and 89–91) [1,2]. Of these, EV71 causes aseptic meningitis, encephalomyelitis, and acute flaccid paralysis, leading to death [3,4]. EV71 is a single-stranded and positive-sense RNA virus. The viral genome is approximately 7,500 nucleotides in length, with a single open reading frame that encodes a large precursor protein [5]. Upon infection, the internal ribosome entry site (IRES) element in the 5’-untranslated region (UTR) initiates the translation of the viral processor polyprotein, which is then proteolytically cleaved by viral 2A, 3C and 3D proteases to form four structural (VP1, VP2, VP3, and VP4) and seven nonstructural (2A, 2B, 2C, 3A, 3B, 3C, and 3D) proteins [6].

Viral infections are treated with vaccines, anti-viral synthetic drugs, and exogenous cytokines [7-10]. Due to multiple viral species and serotype, antigenic variation, and genomic mutations, there is no proven effective treatment for EV71 infection [11]. CHA is the major active ingredient found in many traditional Chinese herbs and herbal medicine [12]. Structurally, CHA is an ester formed between caffeic acid and L-quinic acid. Both CHA and caffeic acid have been reported to have multi-antiviral activities against HIV [13], adenovirus [14], HSV-1, and HSV-2 [15,16]. Although the antiviral activities of CHA have been well studied, little is known about the antiviral effects of CHA on EV71.

In the present study, we examined the effects of CHA on inhibiting EV71 replication. The results demonstrated that CHA can effectively protect from EV71-infected RD cells and inhibit the secretions of IL-6, TNF-α, IFN-γ, and MCP-1. In addition, CHA has antiviral function by interfering with 2A transcription and translation during early-stage EV71 replication.

## Materials and Methods

### Chemicals and RD cells

Chlorogenic acid (C_16_H_18_O_9_; MW 354.3; ≥ 98% pure) were purchased from Aladdin Co. (Shanghai, China). Ribavirin was from Ryan Pharmaceutical Co. (Xuzhou, China). RD cells were purchased from the Chinese Academy of Sciences, Cell Bank of Type Culture Collection (Shanghai, China). DMSO was from Borunlaite Co. (Beijing, China)

#### Virus isolation and propagation

EV71 strain GDV083 (ATCC VR-784) were provided by the China Center for Type Culture Collection (CCTCC) at an MOI of 5 in 4 ml of viral inoculum diluted with maintenance medium. RD cells were grown in high glucose Dulbecco’s modified Eagle’s medium (DMEM) supplemented with 10% fetal bovine serum (FBS, Gibco) at 37 °C in a humidified incubator with 5% CO_2_. When cells reached 90% confluency, the medium was removed and the monolayer cells were washed once with PBS. Approximately 1 × 10^6^ RD cells were incubated with EV71 at a MOI (Multiplicit of infection) of 5 or as indicated and allowed to absorb for 2 h at 37 °C. Unbounded viruses were removed by washing the cells with medium, and 15 ml of maintenance medium was added. Infected cells and culture supernatants were collected at different time intervals. Virus was propagated on 90% confluent cell monolayer in MEM with 2% FCS and antibiotics as described above. Viral titer was determined by cytopathic effect (CPE) and expressed as 50% tissue culture infective dose (TCID50) per ml.

### Cytotoxicity assay of CHA

RD cells (3×10^5^ cells/well) were seeded on 96-well plates and incubated overnight at 37 °C under 5% CO_2_. The medium was removed and different concentrations (160,80, 40,20,10,5 and 2.5 µg/ml) of CHA were added in triplicate. After 2 days’ incubation, the cytotoxicity of CHA was determined by MTT-based method according to the manufacturer’s instructions. The 50% cytotoxic concentration (CC50) of CHA was calculated by regression analysis of the dose–response curve generated from the data.

### Plaque reduction assay

The dose-dependent plaque reduction assay was performed using 2-fold dilutions of CHA in triplicate. Untreated and treated RD cells (3 × 10^5^/well) were infected with EV71 at a MOI of 5, and CHA was added to the cells at varying concentrations. The viruses were absorbed for 1 h at 37 °C, and 1% methylcellulose was added to each well. After 5 days, the viral plaques formed in RD cells were counted by crystal violet staining. The activities of CHA for inhibition of plaque formation were calculated. The 50% inhibitory concentration (IC50) of CHA was the concentration required for 50% inhibition of the indicated virus plaques.

### Effects of CHA on EV71 absorbation and time course analysis

RD cells (5×10^6^) were infected with EV71 in the presence or absence of CHA (20 µg/ml) and ribavirin (40 µg/ml) at a MOI of 5. CHA and ribavirin were added with EV71 at the same time or absorbation for 1 h. Cell supernatants were collected at 0, 4, 8,12, 16, 20, 24, 28, 32 and 36 h postinfection (p.i.), and EV71 titers were determined by a plaque forming assay. In addition, the antiviral activity of CHA was also examined at different time periods before and after viral infection. Briefly, cells (3×10^5^ cells/well) were seeded and incubated for 24 h. CHA (20µg/ml) was added with EV71 at the same time as EV71 (0 h p.i.) or added to the cultures at 2, 4, 6, 8,10, 12, 16, and 24 h p.i. The cell supernatants were collected at 25 h p.i., and a plaque forming assay was performed.

### Reverse Transcription–PCR

Viral RNA was extracted from culture supernatants using QIAamp viral RNA Mini kit (QIAGEN, Germany), and the cDNA was generated in a 20 µL reaction volume for 25 min at 40 °C using random primers and SuperScript II reverse transcriptase (Invitrogen, USA) according to the manufacturer’s protocol. The primers of EV71 VP1, 2A, 3C and 3D were designed by Sangon Biotech Co. (Shanghai, China) and are as follows:

EV71/ VP1: Sense : 5’- TGGCAGATGTGATTGAGAG-3’; Anti-sense : 5’-GGCTTGAAGTGCTGGTA-3’.137 bp. EV71/2A: Sense : 5’-ACCTTAGGGTAGTAAACAGACAC-3’; Anti-sense: 5’-GCAAGCATAAGATGGGACT-3’.262 bp. EV71/3C: Sense : 5’-GACCATCTGGGTAGAGCAT-3’; Anti-sense : 5’-CTTCTGGGATAAACTTGGTG-3’. 143 bp. EV71/3D: Sense : 5’-GGAAGTCTCGCCTGATTG-3’; Anti-sense : 5’-CAGCAGGAGTCATAGTCAGC-3’. 478 bp. GAPDH: Sense : 5’-GGATTTGGTCGTATTGGG-3’; Anti-sense : 5’- GGAAGATGGTGATGGGATT-3’. 205 bp. The PCR fluorescence probing assay reagent (DaAn Gene, China) was commercially available. The cycling conditions were composed of 5 min at 94 °C, followed by 40 cycles with 94 °C for 30 s, 55 °C for 30 s and 72 °C for 1 min, and a final extension cycle at 72 °C for 5 min. The products were visualized on 2% agarose gels containing ethidium bromide and were then sequenced to confirm the amplified band.

#### Cell extracts preparation and western blot analysis

EV71-infected cells were harvested as indicated. The pellet was washed and lysed with a lysis buffer (2% sodium dodecyl sulfate, 35 mM β-mercaptoethanol, 50 mM Tris-HCl [pH 6.8], 1 mM phenylmethylsulfonyl fluoride). Cell lysates were obtained by centrifugation at 13000 rpm and 4 °C. Total protein concentration was determined by the bicinchoninic acid protein assay kit (Pierce). The proteins were then resolved by sodium dodecyl sulfate polyacrylamide gel electrophoresis (SDS-PAGE), and transferred to PVDF membranes (Millipore, USA). Membranes were blocked for 2 h with 5% nonfat dry milk solution in Tris-buffered saline containing 0.1% Tween-20, which were blotted with VP1 / 3D (Bioss Biotech, Beijing, China) and 2A / 3C specific primary antibodies granted by Prof. Lianfeng Zhang (The institute of Laboratory Animal Science, Chinese Academy of Medical Sciences, Beijing, China), and followed by incubation with secondary antibodies conjugated with horseradish peroxidase (Proteintech, USA). The immunoreactive bands were detected by ECL reagents (Pierce, USA) and developed by Super RX film (Fujifilm).

### Detection of inflammatory cytokines

RD cells were infected with EV71 at a MOI of 5 for 1 h at 37°C,and then washed 2 times and cultured in RPMI medium. Supernatants collected at different time points were clarified by centrifugation (13000 rpm). The concentrations of IL-2, IL-6, IL-10, TNF-α, IFN-γ and MCP-1 in culture supernatants from control, EV71 infection, and CHA treatment were analyzed with the luminex fluorescent technique, utilizing Milliplex magnetic beads (Millipore, Billerica, MA, USA) according to the manufacturers’ protocol. The levels of fluorescence in each standard, quality control, and sample were detected with the FLEXMAP3D (Luminex Co., TX, USA). Data was subsequently analyzed using the MILLIPLEX^TM^ Analyst V5.1（VigeneTech Inc., Carlisle, MA, USA). Cytokine concentrations were obtained by interpolation of the standard curve using stepwise five-fold dilution of protein standards. Standard curves were generated for each analyte with the Bio-plex manager software and sample concentrations were calculated from the standard curve.

### Statistics

All data were presented as mean ± S.D. Statistical difference was determined by ANOVA or Student’s t-test as indicated in the figures and legends. A significant difference was considered as *p* value of less than 0.05.

## Results

### Cytotoxic effects of CHA

**Figure 1:**
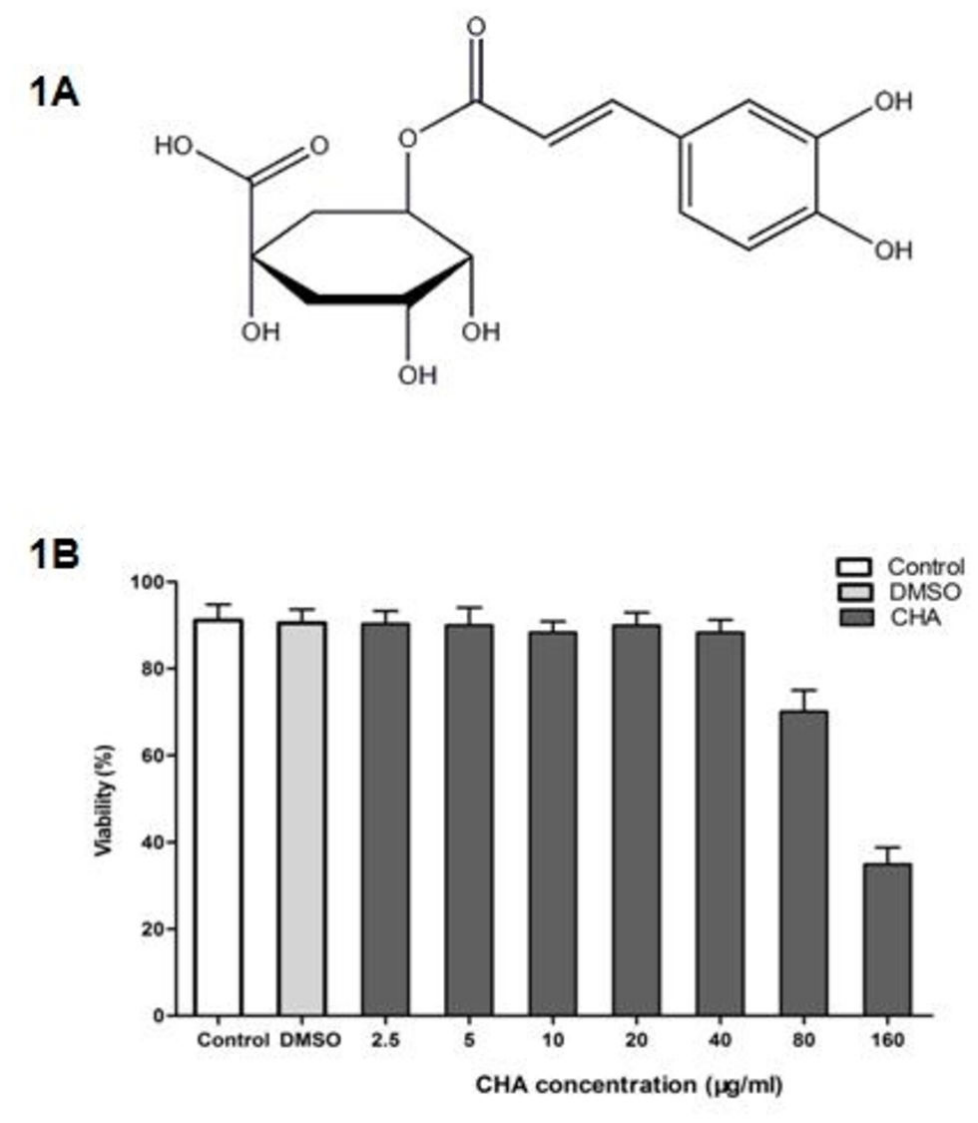
CHA structure and its effect on RD cell viability. (A) The structure of CHA. (B) Control: control ; DMSO: 0.1% DMSO was added in RD cells. CHA was serially diluted as different concentrations (160,80,40,20,10,5 and 2.5 µg/mL) in triplicate. After 2 days of incubation, the cytotoxicity of CHA was determined by MTT assay. Data were presented as mean ± S.D. of three independent experiments.

The molecular structure of CHA is shown in Figure 1A. To delineate whether the inhibitory effects of CHA on EV71 replication was associated with cytotoxicity, we examined the viability of RD cells after treating with CHA for 5 days. Mock treatment with DMSO did not affect cell viability. Compared to control-treated cells, CHA did not show any cytotoxic effects against RD cells at concentrations up to 40 µg/ml (Figure 1B). These results demonstrated that inhibitory mechanisms of CHA on EV71 replication were not cytotoxic. The IC50 of CHA was 121.5 µg/ml.

### Antiviral effects of CHA against EV71

**Figure 2:**
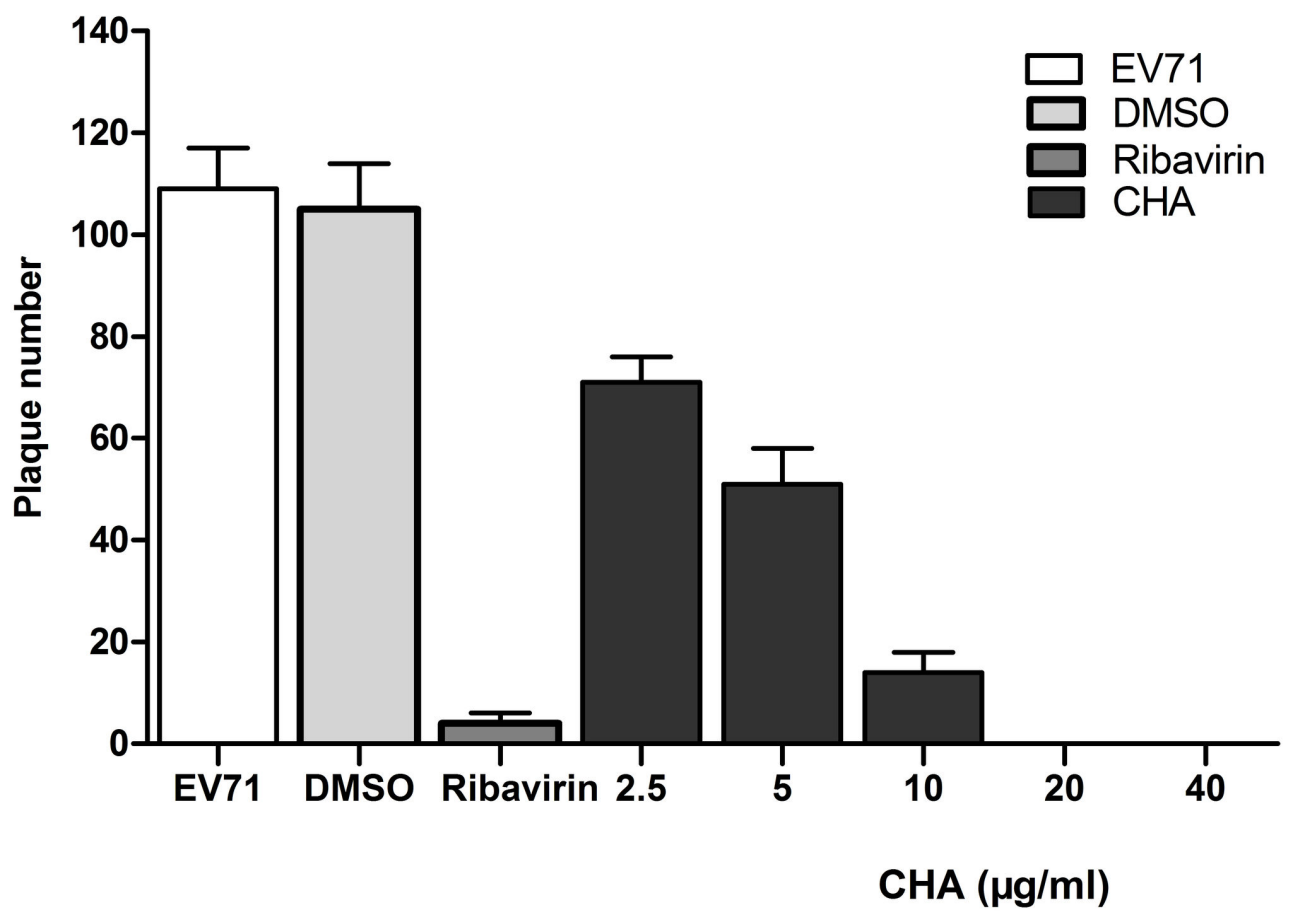
Effects of CHA on EV71 replication. Ribavirin was used as positive control and 0.1% DMSO as negative control. Inhibitory effects of ribavirin and indicated concentration of CHA on EV71 replication were determined by a plaque reduction assay. Data were represented as mean ± S.D. of three independent experiments.

Our results showed that CHA and ribavirin could significantly inhibit the cytopathic effect of EV71. In a plaque reduction assay, CHA-induced inhibitory effects of CHA on EV71 replication was concentration-dependent (< 40 µg/ml). At 2.5, 5, 10, 20, and 40µg/ml, the inhibitory rates of CHA were 34.9 ± 4.6, 53.2 ± 6.4, 87.1 ± 3.7, 100 ± 0.0, and 100 ± 0.0%, respectively, with an IC50 value of 6.3 µg/ml. However, the inhibitory rate of ribavirin with 40 µg/ml was 96.3 ± 1.8% (Figure 2).

### Effects of CHA on EV71 absorbation

**Figure 3:**
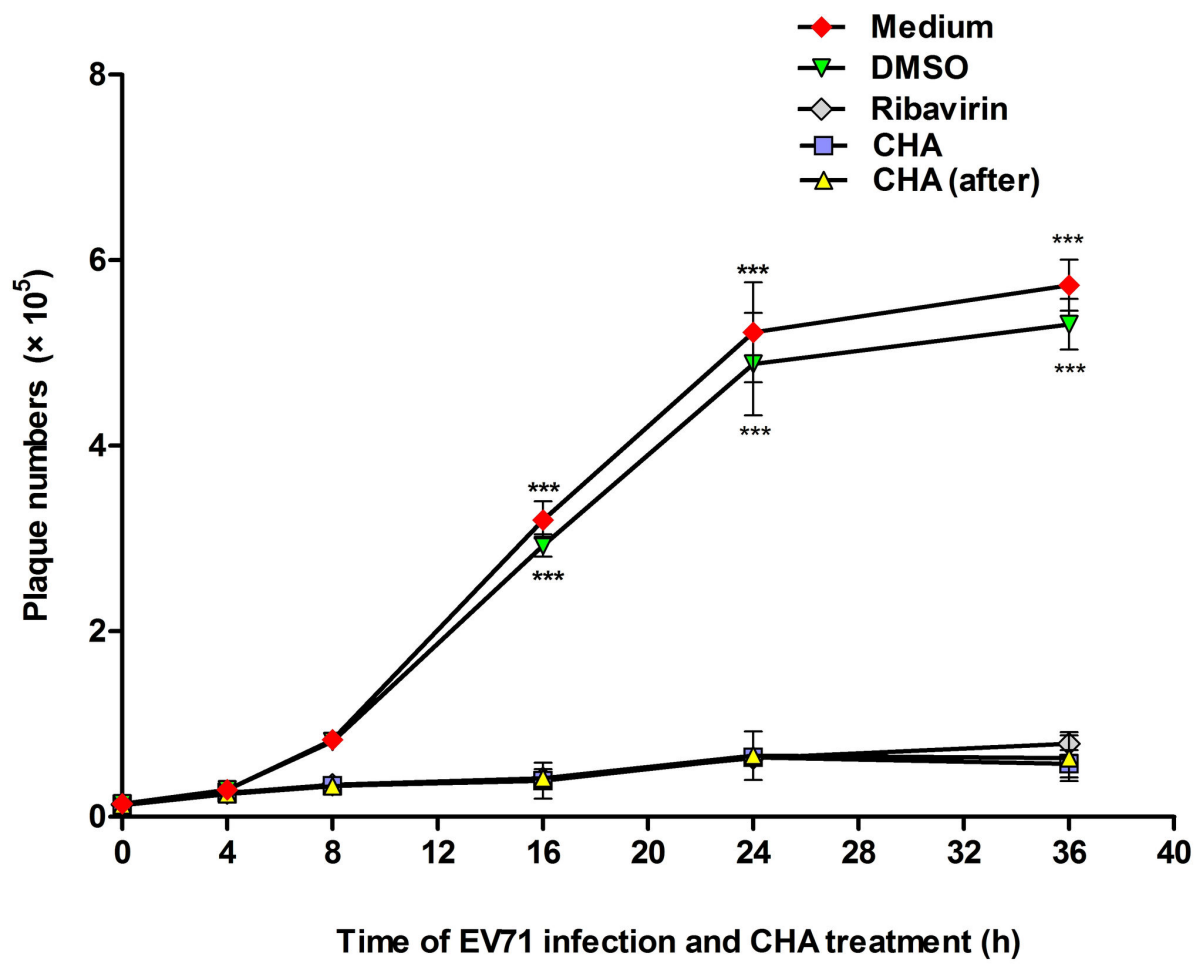
CHA inhibits EV71 replication in RD cells. RD cells (5×10^6^) were infected with EV71 at a MOI of 5 in the presence or absence of CHA (20 µg/ml) and ribavirin (40 µg/ml). CHA and ribavirin were added with EV71 at the same time or absorbation for 1 h. Then cell supernatants were collected at 0, 4, 8,12, 16, 20, 24,28, 32 and 36 h p.i. and the viral titers were determined by a plaque forming assay. Each point represents the mean ± S.D. of three independent experiments (*** *p* < 0.001).

Cell supernatants were collected at 0, 4, 8, 12, 16, 20, 24, 28, 32 and 36 h p.i., and EV71 titers were determined by a plaque forming assay. The viral titers in cell supernatants gradually increased at 8 h p.i. and the titers peaked at 36 h p.i. DMSO did not affect EV71 propagation. However, when CHA (20 µg/ml) and ribavirin (40 µg/ml) were added at the same time as EV71 or after EV71 absorbation for 1 h, the viral titers in cell supernatants decreased (Figure 3). These results demonstrated that CHA could block EV71 replication in RD cells, which might be independent of inhibiting viral absorbation.

### Time course analysis of the effect of CHA on EV71 replication

**Figure 4:**
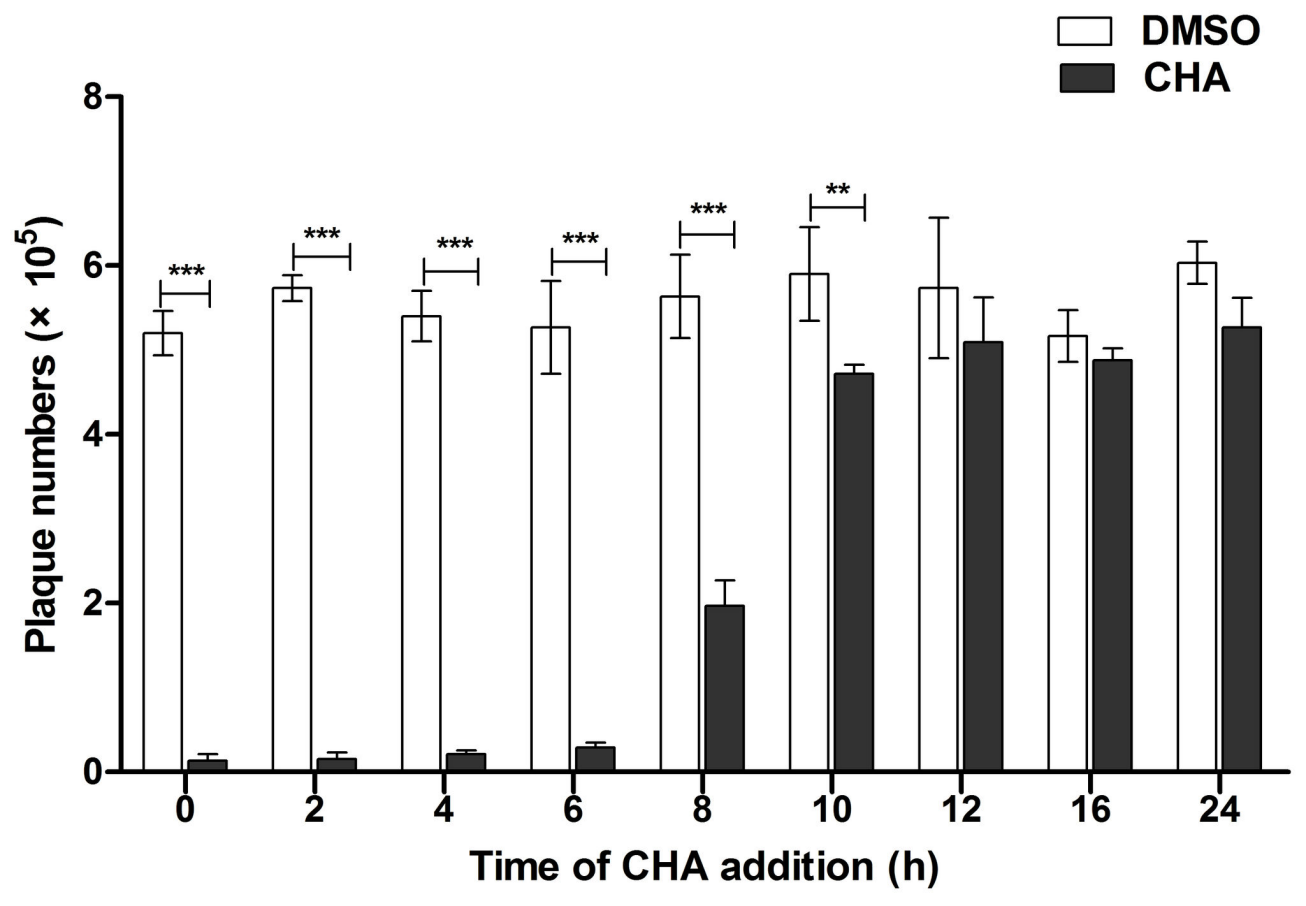
Effects of CHA on EV71 replication before or after viral infection. DMSO: EV71 infection with DMSO. CHA: EV71 infection with DMSO and CHA RD cells (5 × 10^6^) were infected with EV71 (5 MOI) and CHA (20 µg/ml) was added at the indicated time. Cell supernatants were collected at 25 h p.i. and EV71 titers were determined by a plaque forming assay. Each bar represents the mean ± S.D. of three independent experiments（** *p* < 0.01, *** *p* < 0.001).

Time course experiments were performed to determine at what point CHA inhibited replication of EV71. CHA (20 µg/ml) was added with EV71 (0 h p.i.) or added to the cultures at 2, 4, 6, 8, 10, 12, 16, and 24 h p.i. The cell supernatants were collected at 25 h p.i., and a plaque forming assay was performed. The results indicated that the addition of CHA between 0 and 10 h significantly suppressed EV71 replication (Figure 4). Addition of CHA at 12-24 h p.i. showed only partial or slight inhibitory effects on EV71 replication. Therefore, the inhibitory effects of CHA might be related to the blocking of EV71 gene expression necessary for its replication.

### CHA reduces EV71 2A mRNA synthesis

**Figure 5:**
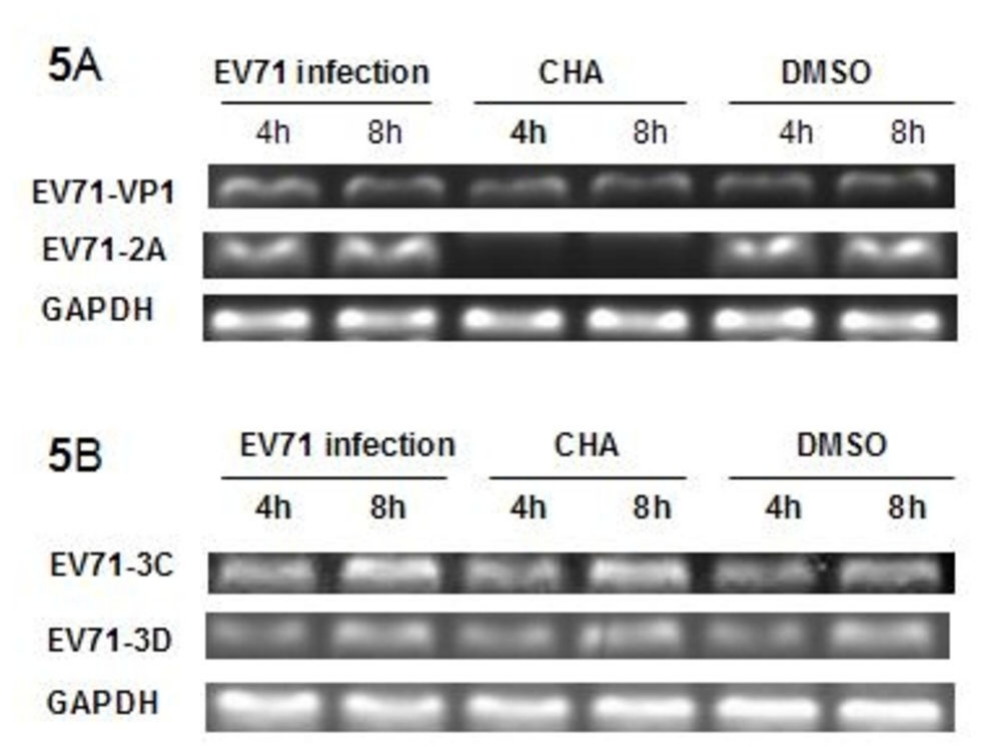
Effects of CHA on EV71 mRNA synthesis in RD cells by RT-PCR. EV71 infection: CHA treatment: EV71 infection in the presence of CHA. DMSO: EV71 infection treated with 0.1% DMSO. RD cells were infected with or without 5 MOI of EV71 in the presence or absence of CHA at 20 µg/ml. Total RNA was extracted from RD cells at 4 h and 8 h p.i. The amplified products were subjected to 2% agarose gels.

We next assessed whether CHA interferes with the effect of VP1, 2A, 3C, and 3D proteases in RD cells. The experiments were divided into three groups, including EV71 infection group, CHA treatment group, and DMSO control group. At least 5 × 10^6^ cells were infected with EV71 (MOI = 5) in the presence or absence of CHA at 20 µg/ml. The expression of VP1, 3C, and 3D were found to be robust in cell lysates harvested at 4h and 8h p.i. among EV71 infection, and CHA and DMSO treatment. At 20 µg/ml CHA, the mRNA level of *EV71* 2A was greatly reduced, and its expression was hardly detected at 4 and 8h p.i. (Figure 5). The results suggested that CHA can block viral RNA synthesis at early stages in EV71-infected RD cells.

### CHA inhibits the expression of EV71 2A proteinase

**Figure 6:**
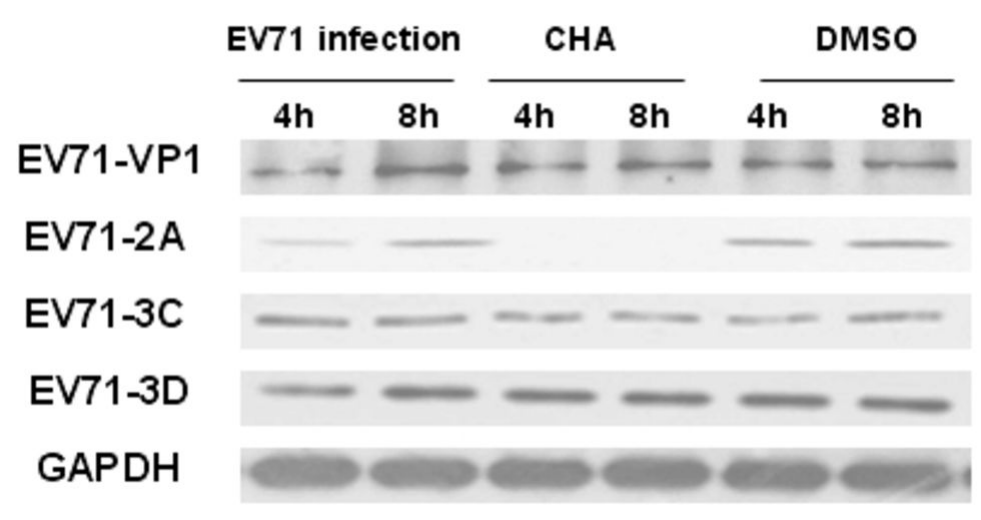
Effects of CHA on EV71 protein expression. EV71 infection: RD cell-infected with EV71 (MOI = 5). CHA treatment: EV71 infection in the presence of CHA (20 µg/ml). DMSO: EV71 infection treated with 0.1% DMSO. At 4 and 8h, the cell lysates were subjected to 10% SDS-PAGE and transferred to PVDF membrane to determine the level of EV71 VP1, 2A, 3C, and 3D.

To further analyze whether CHA can interfere EV71 protein translation, 5 × 10^6^ cells of RD cell-infected with EV71 (MOI = 5) were treated with or without CHA (20 µg/ml). Total cellular protein was extracted at 4h and 8h p.i., and EV71 VP1, 2A, 3C and 3D proteins were detected by western-blotting. The results indicated that the expressions of EV71 VP1, 3C and 3D protein increased among the three groups. However, the expression of EV71 2A protein was significantly reduced in the presence of CHA at 20 µg/ml at 4 and 8 h p.i. (Figure 6). The results suggested that CHA can inhibit EV71 2A protein synthesis at early stages in EV71-infected RD cells.

### EV71 infection stimulated cytokine secretion

**Figure 7:**
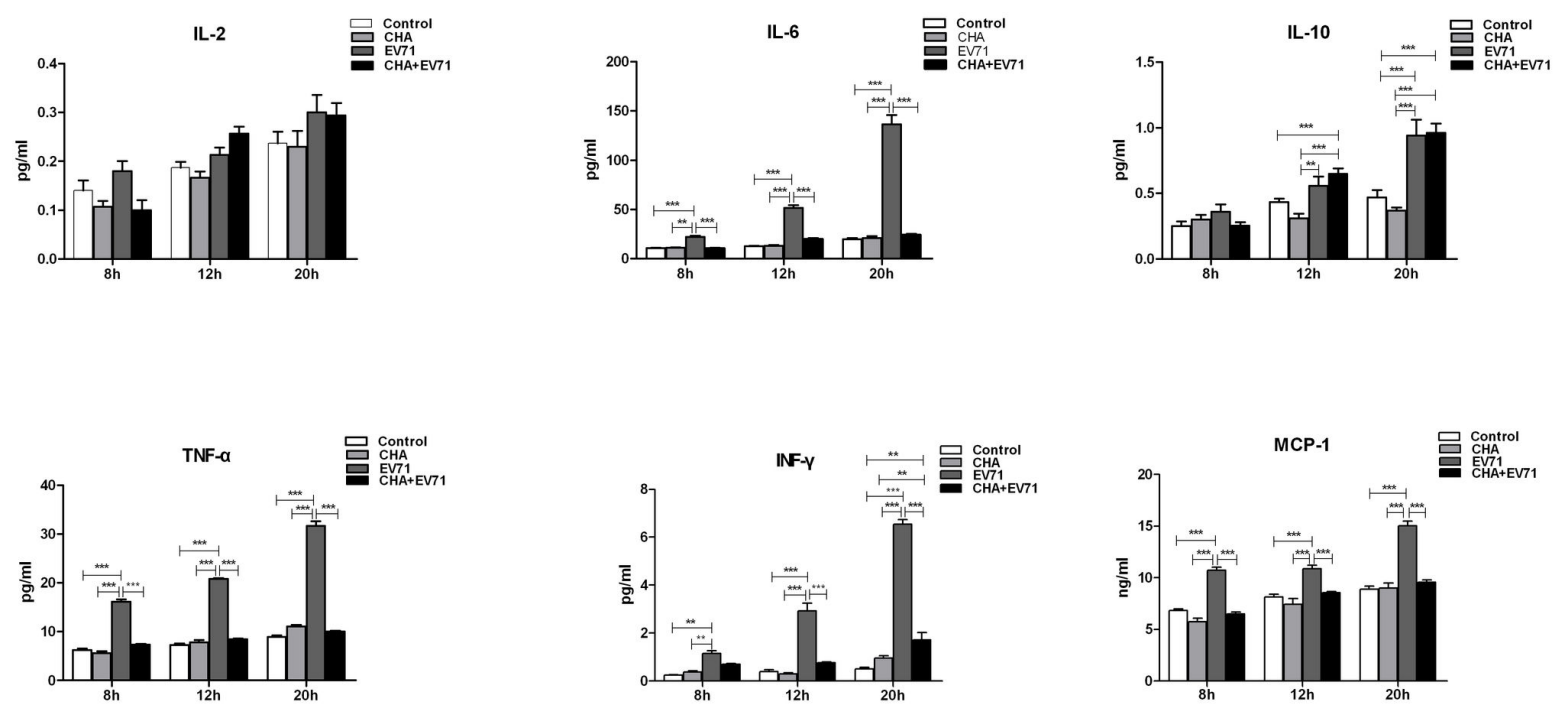
EV71 infection stimulates cytokine secretion. Control: Uninfected RD cells; EV71: RD cell-infected with EV71 (MOI = 5); CHA: EV71 infection in the presence of CHA (20 µg/ml). RD cells were absorbed with EV71 for 1h, and then treated with or without CHA(20 µg/ml). Culture supernatants were collected at 8h, 12h, and 20h. The concentrations of IL-2, IL-6, IL-10, TNF-α, IFN-γ and MCP-1 were detected by luminex fluorescent technique. The data were expressed as mean ± SE from 3 independent experiments. ** *P* < 0.01 and ***P < 0.001 by two-way ANOVA.

RD cells were infected with EV71 at a MOI of 5 for 20 h. Compared to the control, EV71-infected cells had increased IL-6, IL-10, TNF-α, IFN-γ, and MCP-1 production at 8 h, 12 h and 20 h p.i. However, the levels of IL-2 did not changed between control and EV71-infected cells. CHA treatment (20 µg/ml) decreased IL-6, TNF-α, IFN-γ and MCP-1 secretion in EV71-infected RD cells. Therefore, CHA could effectively inhibit inflammation to mitigate damage of RD cells (Figure 7).

## Discussion

Several stages of the viral life cycle are therapeutically targetable as antiviral agents. Virus-encoded proteases and polymerases have been the most prominent targets for antiviral drug development [17,18]. However, effective antiviral agents and vaccines against EV71 are currently still under development [19].

EV71 is a genetically diverse and rapidly evolving virus [20,21]. Viral mutation with drug resistance could develop soon after chemotherapy. Traditional Chinese herbs have been reported to be effective for treating EV71 infection [22-24]. CHA is an important plant polyphenol widely distributed in the leaves and fruits of dicotyledonous plants such as Flos Lonicerae Japonicas and coffee bean. Previous studies reported that CHA showed a variety of biological effects, including anti-inflammatory, antioxidant, neuroprotective activity, and inhibition of HSV [25,26], HIV-1 [27], and HBV replication [28]. However, it is still unknown whether CHA is effective against EV71. Our studies demonstrated that CHA did not show any cytotoxicity against RD cells at concentrations up to 40 µg/ml, and the IC50 of CHA was at 121.5 µg/ml. Ribavirin is a guanosine (ribonucleic) analog and nucleoside inhibitor used to stop viral RNA synthesis and viral mRNA capping [29,30]. Our results showed that both CHA and ribavirin could significantly inhibit the cytopathic effect of EV71.

The plaque reduction assay indicated that the inhibitory effect of CHA on EV71 replication is concentration-dependent and its IC50 was 6.3 µg/ml. When both CHA (20 µg/ml) and ribavirin (40 µg/ml) were added simultaneously with or after EV71 for 1 h, the viral titers in cell supernatants were decreased. These results demonstrated that CHA could block EV71 replication in RD cells, which might be independent of its effects against viral attachment and penetration. Time course experiments showed that the addition of CHA between 0 and 10 h significantly suppressed EV71 replication, whereas there were only partial or slight inhibitory effects on EV71 replication at 12–24 h p.i. Therefore, the inhibitory effects of CHA might be related to blocking EV71 expression during early-staged EV71 replication.

EV71 has a single-stranded positive-sense RNA encoding four capsid proteins and seven nonstructural protein, which are arranged in the order of NH2-VP4-VP2-VP3-VP1-2A-2B-2C-3AVPg-3C-3D-COOH. In addition to protecting the viral RNA from nuclease cleavage, the capsid proteins VP1, VP2, and VP3 are antigenic and recognize the receptors on the surface of specific host cells. The nonstructural genes are involved in processing the viral genome, promoting viral replication, and shutting down the production of host cell proteins. In particular, 2A, 3C, and 3D proteins perform multiple enzymatic functions [31]. EV71 2A is a cysteine protease that cleaves the eukaryotic initiation factor 4GI, a key factor for host protein synthesis [32]. Moreover, transient expression of EV71 2A protease alone also induced apoptosis [33,34]. In the presence or absence of CHA at 20 µg/ml, the gene expressions of EV71 VP1, 3C and 3D were strongly induced at 4 and 8 h p.i. after EV71 infection and CHA treatment. To our surprise, the mRNA level of EV71 2A was greatly reduced at 4 and 8h p.i. The results suggested that CHA can block viral RNA synthesis at early stages in EV71-infected RD cells. The results indicated that the expressions of EV71 VP1, 3C and 3D protein increased among the three groups. However, the expression of EV71 2A protein was significantly reduced in the presence of CHA at 20 µg/ml at 4 and 8h p.i., respectively. This suggested that CHA can inhibit EV71 2A protein synthesis at early stages in EV71-infected RD cells.

Several proinflammatory cytokines (ie. IL-1, IL-6, and TNF-α) are induced by oxidant stress, cytokines, and viral infection. These insults leads to host cell damages, chronic inflammation, and other immunoresponses [35-37]. In this study, EV71 infection could increase production of IL-6, IL-10, TNF-α, IFN-γ and MCP-1 at 8 h, 12 h and 20 h p.i. However, the levels of IL-2 were unchanged in EV71-infected RD cells. The secretions of IL-6, TNF-α, IFN-γ and MCP-1 in EV71-infected RD cells were decreased after CHA treatment. Therefore, CHA could effectively inhibit inflammatory reaction and mitigate RD cell damages.

In summary, CHA was effective to inhibit EV71 infection and replication through targeting the early stages of the viral life cycle after entry. This was mediated by a decrease in IL-6, TNF-α, IFN-γ and MCP-1 secretion. Therefore, CHA is a potential drug for treating EV71 infection.
